# RETRACTED: Xiao et al. TTF1, in the Form of Nanoparticles, Inhibits Angiogenesis, Cell Migration and Cell Invasion In Vitro and In Vivo in Human Hepatoma through STAT3 Regulation. *Molecules* 2016, *21*, 1507

**DOI:** 10.3390/molecules31020386

**Published:** 2026-01-22

**Authors:** Bin Xiao, Dongjing Lin, Xuan Zhang, Meilan Zhang, Xuewu Zhang

**Affiliations:** 1 College of Medicine, Yanbian University, Yanji 133000, China; binniuniu@163.com (B.X.); zhxzk1028@126.com (X.Z.); shaokuiyang@163.com (M.Z.); 2 Basic Medical College, Jilin Medical University, Jilin 132013, China; zupei2628@163.com

The journal retracts the article titled, “TTF1, in the Form of Nanoparticles, Inhibits Angiogenesis, Cell Migration and Cell Invasion In Vitro and In Vivo in Human Hepatoma through STAT3 Regulation” [1], cited above.

Following publication, concerns were brought to the attention of the Editorial Office regarding the integrity of a number of figures presented in this article [1].

In accordance with standard journal procedures, the Editorial Office and Editorial Board conducted an investigation, which confirmed duplications in Figures 2, 4, 5 and 6. Despite multiple attempts, the authors failed to respond or adequately address these concerns. Consequently, the Editorial Board has lost confidence in the reliability of the findings and has decided to retract this publication [1], as per MDPI’s retraction policy.

This retraction was approved by the Editor-in-Chief of the *Molecules* journal.

The authors have been informed of this retraction but did not provide a comment on this decision.
